# Using immersive technologies to facilitate location scouting in audiovisual media production: a user requirements study and proposed framework

**DOI:** 10.1007/s11042-022-13680-8

**Published:** 2022-09-14

**Authors:** Aimone Bodini, Federico Colecchia, Arthi Manohar, David Harrison, Vanja Garaj

**Affiliations:** Brunel Design School, Brunel University London, Kingston Lane, Uxbridge, Greater London, UB8 3PH UK

**Keywords:** Audiovisual production, Real-time graphic engines, Virtual reality, Augmented reality, Virtual production, Location scouting

## Abstract

In common with many industries, the audiovisual sector is likely to be transformed by real-time graphic engines in combination with immersive technologies such as Virtual Reality and Augmented Reality. This technological mix enables what is presently known as Virtual Production, introducing a new process to the audiovisual professional, capable of fostering creativity, collaboration, and decision making, while at the same time increasing efficiency. The potential for Virtual Production to transform workflows and creative processes within large-scale productions in the audiovisual sector is significant and includes the prospective introduction of new capabilities for remote co-creation of content. However, barriers to democratisation need to be overcome, particularly in relation to adoption of the technology by small and medium independent productions that generally have limited access to resources and technical knowledge. Following an extensive study of the current literature and involvement in the research of professionals working in the field through online interviews, this article documents a first step towards filling this gap by investigating Virtual Production adoption scenarios for small and medium independent productions. The primary aim is to design intuitive, engaging, and effective solutions to address the needs of Directors, Cinematographers, and Producers.

Thanks to the valuable time and experience of industry professionals, this study has gathered user requirements for a Virtual Production design solution capable of facilitating location scouting and pre-production. A novel framework for remote exploration of target locations within immersive environments, co-created with relevant stakeholders, is presented to lay the foundations of future co-design work.

## Introduction

The audiovisual (AV) industry has been reshaped by social and cultural changes, as well as by the introduction of new technologies and processes including Virtual Production (VP). Different players in the industry and academic researchers [12, 16, 19] agree that VP can be described as the process of pre-producing, producing, and post-producing AV content using real-time computer graphic (CG) engines such as Unity [40] and Unreal Engine [13]. This approach has shifted the traditional linear workflow to a non-linear one where it is possible to perform quick iterations and explore different creative decisions.

“Virtual production is where the physical and digital worlds meet.”

— Weta Digital qtd. in [19]

Until recent years, adopting the VP process required high-end technical resources and highly-skilled professionals with years of experience in digital Visual Effects (VFX) and the real-time engine industry [12]. Therefore, it is not a coincidence that the first AV production incorporating VP was the blockbuster movie ‘Avatar’ (2009) [37]. Today, thanks to the continuous advancements and democratisation of the technologies that support it, the VP process is becoming common practice that brings creative and economic benefits [30] both to the high-end productions such as ‘The Mandalorian’ (2019) [31] and to independent productions such as ‘I Am Mother’ (2019) [20].

### Benefits of virtual production

**Fig. 1:**
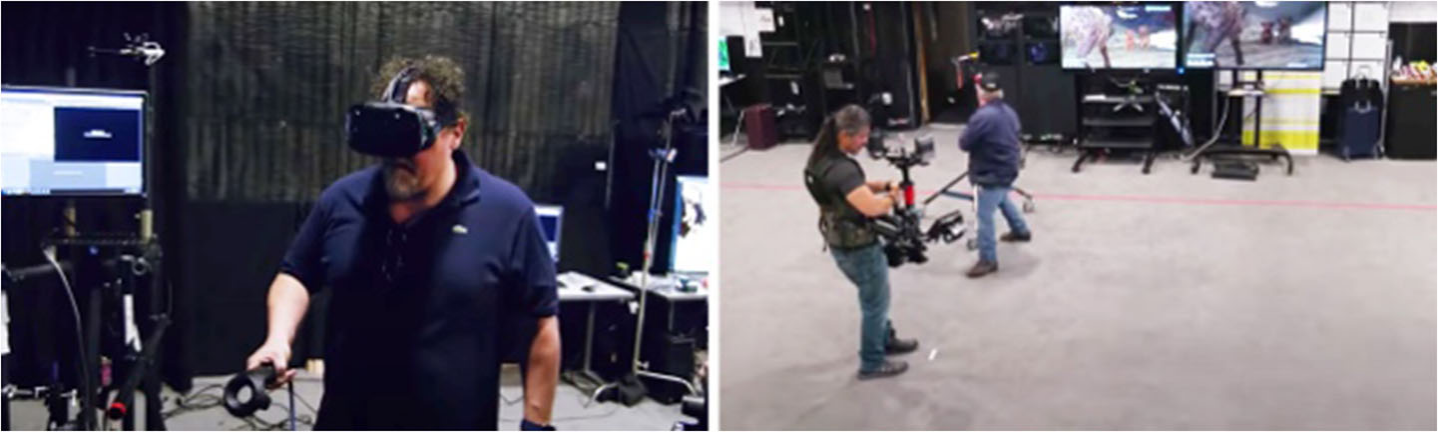
The ‘Lion King’ Director Jon Favreau working in VR (left). Steadicam operator shooting the virtual scene within an empty room equipped with sensors and computers to track physical camera movements in real time (right). Images extracted from Youtube channel [4]

A common need for many AV productions is in-camera pre-visualisation of the VFX that will later be integrated during the post-production phase with specific 3D and VFX software [9]. Because real-time graphic engines are at the core of the VP process, it is possible for the Director and his collaborators to see on the set, in real time, how the VFX they plan to integrate will most likely look once post-produced. Thanks to this unpolished but plausible representation of the VFX, the required adjustments can be made in order to achieve the picture envisioned for shooting, rather than waiting for weeks or even months to see the result of such integration. This process can drastically reduce the time to make decisions and allow for the creative team to explore different ideas more efficiently and therefore facilitate their iteration. Another objective for those productions aiming to recreate imaginary worlds is scouting of fictional locations that do not exist in reality, but which will function as a canvas for the story. This subprocess of VP is called ‘Virtual Location Scouting’. An example is given from the making of ‘The Lion King’ (2019) [14], a full CG feature film, whereby the immersive technologies integrated in the VP process proved to be crucial. In the pre-production stage of this film, the project virtual art department procedurally recreated an artificial version of Africa savannah, which could be experienced wearing a Virtual Reality (VR) headset. This model allowed the director and his collaborators to scout the savannah virtually as if it were a real location. It was possible for them to have a 6DoF (6 degrees-of-freedom) navigation within the virtual location, to instantly ‘teleport’ themselves miles away across the savannah, and to employ traditional filmmaking gear including real cameras, tripods, and cranes to frame shot﻿s and s﻿et camera movements (Fig. 1). Recreating this filmmaker-friendly location scouting workflow through the use of VR and real-time graphic engines helped the team decide where and how to shoot the movie scenes.

### Role of location scouting in traditional AV productions

**Fig. 2:**
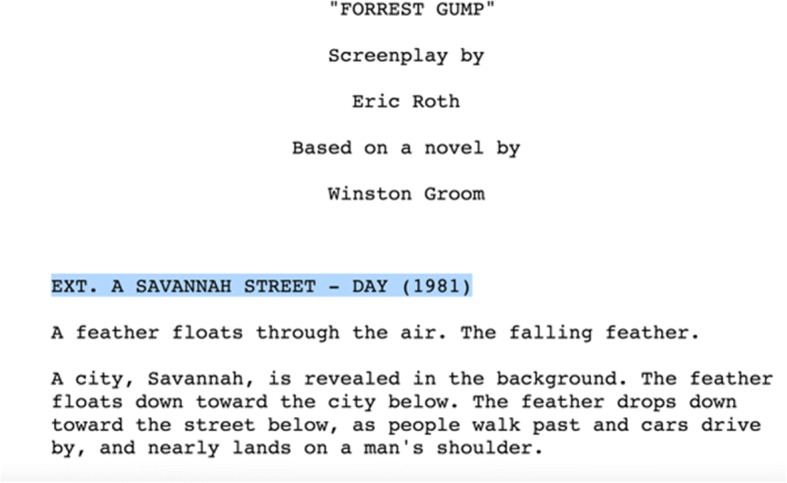
‘Forrest Gump’ Screenplay. From [28]

Location Scouting is one of the main stages in the pre-production phase, which can have a considerable impact on production and post-production. Evaluating the choice of location to shoot in is a crucial decision made by the Production Designer after reading the script. A further proof of the importance of the location choice is given by the fact that the first information given to the reader of a traditional screenplay is where the scene is set (Fig. 2). Deciding the location to set the scene in affects different creative and technical aspects and the production budget. Even once the shooting location has been identified, it is considered useful for some roles such as Director, Cinematographer, Producer, First Assistant Director, and Set Designer to visit that place again and improve the planning of the scene both creatively (e.g. where to place the camera, which camera lens to use and how to light the scene) and technically (e.g. how to reach the location, where to set up the wardrobe and to check which power sources are available).

#### Who is awaiting to be empowered by VP?

The AV industry is currently very fragmented: TV series, feature films, short films, animation, documentaries, sport broadcasting, music videos, and video art are a number of the different segments filmmakers and production companies may operate in. It is important to highlight that not all these productions are set in imaginary worlds, nor do they need to integrate VFX in their shots. Shooting in real-world locations is often considered more appropriate for creative and production reasons. In such a scenario, the following question may be asked: “How can independent AV productions adopt VP to enhance their location scouting and pre-production process when shooting in a real-world location?”. This question is suggested by the already proved advantages of VP in other contexts and production phases [3, 18]. Further relevance to the question is given by the convergence of unprecedented trade-offs in terms of affordability and performance of three key technologies: real-time graphic engines, immersive technologies, and scanning technologies. If such technologies can be integrated within a new VP workflow, it is reasonable to envisage benefits in terms of time savings and use of financial resources, as well as further opportunities for creatives working on independent productions.

### Related work

In recent years, VP has been the subject of several studies investigating its impact on various pre-production, production, and post-production processes. The main focus in the current academic literature is on understanding how filmmakers can be helped when shooting scenes with embedded VFX elements, exploiting VP and the latest advancements in real-time technologies [11, 24]. Researchers at the Filmakademie Baden designed *Virtual Production Editing Tool* (VPET), an Augmented Reality (AR) tool developed for consumer devices such as iPad and iPhones to enable on-set pre-visualisation for independent filmmakers in a relatively affordable and accessible way [34]. One of the main features of VPET is that it enables the so-called ‘set extension’ of part of the film set. This augmentation of a pre-existing scenography enables production designers to visualise on their tablet or smartphone the final look of the scene and to communicate their vision to other departments (e.g. camera and art department). Adopting this solution, it was also possible for the production designer to communicate and present ideas to the Director and Cinematographer more effectively. In addition, AR proved to be useful in a real-world location scenario [42], as it allowed the user to place and manipulate virtual elements (e.g. objects and characters) in the real-world environments (e.g. streets and football fields) that were the planned filming locations. This process enabled the pre-visualisation of the elements, which were not available during pre-production and which were going to be used on shooting day (production phase). VP has also been explored in order to train actors’ performances in a virtual environment before the beginning of production [7], and to facilitate filmmakers while using motion capture technologies for animated productions where actor movements have to be transposed to those of a fictional CG character [5]. Similarly, other studies designed and evaluated the use of VR as a training tool for helping novice filmmakers learn cinematography fundamentals, putting them in the shoes of different film roles such as the Director, Cinematographer, and Editor [22]. The preliminary study documented positive results from the use of the VR system that the researchers developed and how it can be further improved adding more features to simulate the complexity of real camera operation more accurately. Industry players such as The Third Floor developed a proprietary tool called ‘Pathfinder’, designed for virtual location scouting [38]. Such a tool makes it possible to build from scratch a virtual representation of a physical set.

After a critical analysis of both academic studies and industry applications, the investigation that is presented in this article has highlighted a significant knowledge gap in relation to the benefits that VP and immersive technologies can potentially bring to independent AV professionals as opposed to large-scale film studios. As previously shown, other studies have explored how consumer-level immersive devices can be employed within a controlled environment, but only a few have investigated how these new immersive solutions can be applied outside the realm of large-scale film studios. Despite the frequent use of real-world locations by independent productions and the important role that choice of location has in financial and creative terms, little has been done to investigate the benefits that VR, AR, and related technologies can bring to filmmakers. Moreover, in most of the cases presented, when trying to employ new technologies and workflow-enhancing solutions, it has been necessary to bring onboard new people to manage the innovative process [41]. This requirement has an impact on the size and complexity of the production, which is the opposite to what small and agile independent productions should and are able to favour.

Arguably, the ease of use and intuitive design of a new workflow is crucial for VP adoption by independent filmmakers. In addition, it is speculated that such a workflow could be appealing from a financial perspective. As reported by Leipzig [21], despite the small budgets that individual independent productions manage compared to blockbuster productions, the ‘independent’ world is composed by a much larger number of projects. Chris Anderson has popularised the concept of the ‘long tail’, pointing to a totally new business model for media consumption and explaining how niche contents (‘tail’) opposed to hits (‘head’) represent a new and relevant market: *“Combine enough nonhits on the Long Tail and you’ve got a market bigger than the hits”* [2].

## Study

**Fig. 3:**
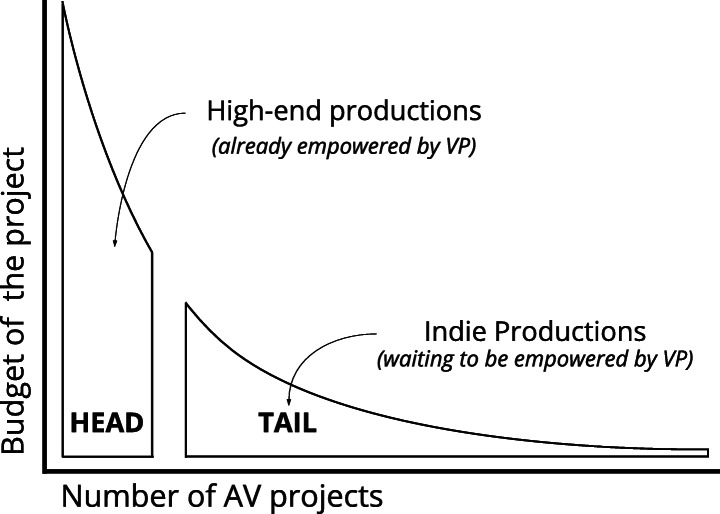
Adaptation of‘ The Long Tail’ graph

**Fig. 4:**
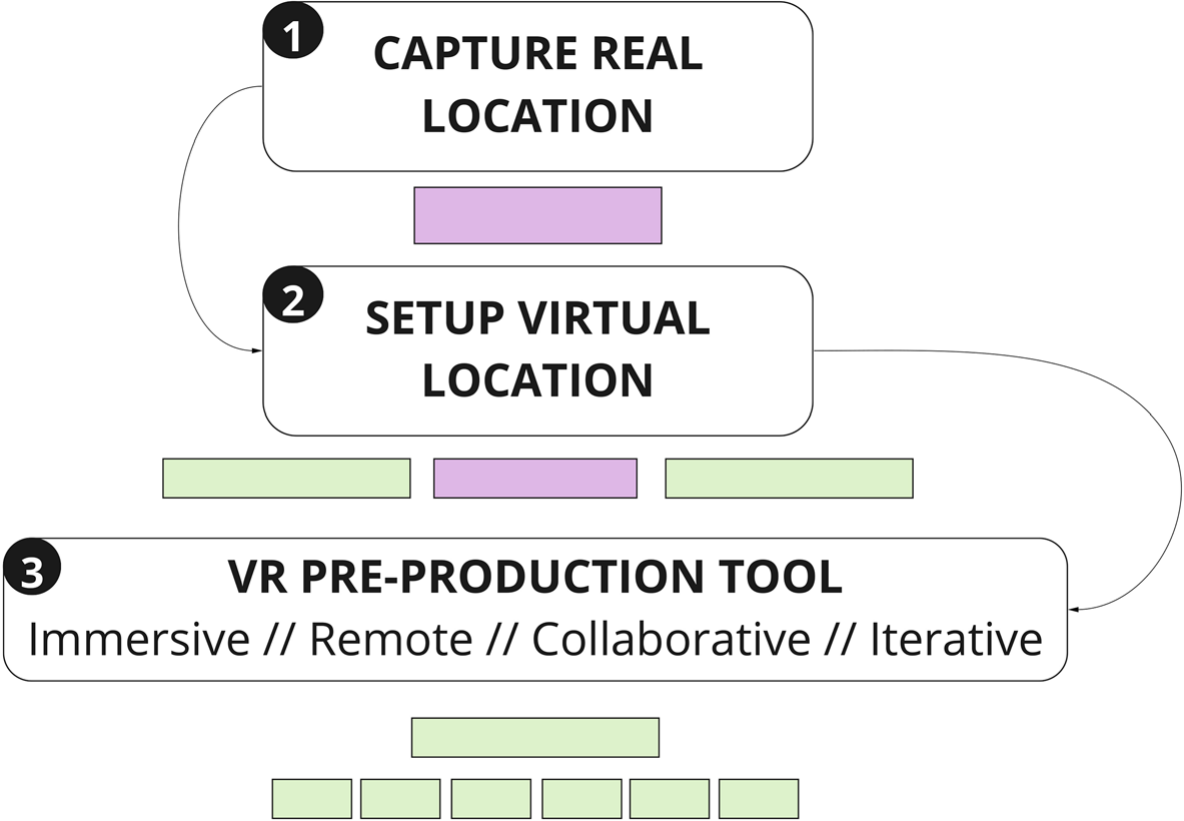
Initial System Design Diagram

This paper reports an exploratory user requirement﻿s study to define the potential benefits that immersive technologies such as VR and AR, real-time graphic engines, and related hardware and software solutions, can bring to independent AV content production professionals, empowering them and facilitating their work during the pre-production phase of live-action projects, with an  emphasis on location scouting. The paper also presents a system diagram for a novel location scouting framework, which was co-created with participants as part of the study proceedings Figs. 3 and 4.

### Method

The reported study has been developed based on participatory design principles. The concept of ‘participatory design’ has been used in the academic community for some time [6, 25] and defined in depth by researchers such as Schuler [29], Simonsen [32], and Spinuzzi [35]. According to Spinuzzi, the primary objectives of participatory design are to i) involve stakeholders in the innovation process, ii) consider their views on an initial design concept, and iii) co-create a new design solution together with the stakeholders. The application of participatory design has been critical to understand further the current approach of AV production professionals to location scouting. Furthermore, it is believed that the professionals’ active participation in the design process can improve not only the location scouting stage as such, but the whole workflow employed by AV productions.

The study involved 20 AV production professionals in semi-structured, one-to-one interviews, which were all conducted online due to COVID-19 restrictions. Establishing how many participants to involve in in-depth interviews is not a trivial decision, and Hennink et al. [17] tried to answer this question based on a study in which they interviewed the total of 25 participants. While conducting the study, they found that a range of thematic issues was identified after interviewing nine participants. Nonetheless, it was only after 16–24 interviews were completed that they developed a rich understanding around the subject matter of the study. Within the context of this study, the saturation of the analysis *codes* was reached after interviewing two thirds of the participants. The last third reinforced and added detail to the previously discussed themes, thereby offering slightly different perspectives on the various topics identified. This addition was helpful not only for taking note of all the issues raised by the stakeholders, but also for gaining a deeper understanding of the problem and its context.

#### Participants

The 20 study participants have all been active in the AV industry at the time of the study, working on a variety of AV projects (feature films, short films, documentaries, commercials, music videos, and video art). The participants were based in the US (9), Canada (1), UK (3), and Italy (7). The majority (85%) were between 25 and 40 years old, a generation referred to as Millennials [36]. Millennials have grown up experiencing digital technology and represent the next generation of filmmakers. Three of the participants were over 40 years of age, two of these being leading VP experts.

The sample of participants involved comprises ‘relevant stakeholders’ (18) and ‘leading experts’ (2). The first group includes those involved in the pre-production phase of a project such as Director and Cinematographer, and operating in small and medium-size productions. Being the potential users of the design solution resulting from this study, they were very well suited to share their current approach to location scouting, comment on the challenges of pre-production, and give a reliable evaluation of the system design diagram of the solution proposed in this article. The participants in the second group are leading VP experts. It was decided to involve them in order to gather more information on state-of-the-art technology and to benefit from their experience of adopting the technology in major blockbuster productions. In addition to providing expert input, the second group commented on the specific stakeholder needs for participation in high-end structured productions. This clarification helped differentiate the needs of stakeholders involved in major as opposed to independent productions. Seven out of 18 participants have worked on projects involving real-time technologies, VR, and AR, and the remaining 11 have a good understanding of the macro-concepts surrounding immersive technologies and how they can be adopted Table 1.

**Table 1:** A summary table of the total participant pool

PARTICIPANT	ROLE	DESCRIPTION
P01	First Assistant Director	Assisting Directors and Producers on daily tasks
P02	Producer	Overseeing the entire process and actively looking for funding
P03	Cinematographer	Operating cameras and lighting on set, working closely with Director
P04	Leading Expert	Designing and overseeing the VP process on blockbuster movies productions
P05	Leading Expert	Designing and overseeing the VP process on blockbuster movies productions
P06	Producer	Overseeing the entire process and actively looking for funding
P07	Producer	Overseeing the entire process of commercials
P08	Director	Directing short-films and commercials
P09	Director	Directing short-films and commercials
P10	Cinematographer	Operating cameras and lighting on set, working closely with Director
P11	Director	Directing short-films and commercials
P12	Cinematographer	Operating cameras and lighting on set, working closely with Director
P13	Director	Directing short-films
P14	Director	Directing short-films
P15	Producer	Overseeing the entire process and actively looking for funding
P16	Director & Producer	Overseeing the production of corporate videos and social media contents
P17	Producer	Involved during the development phase of a project
P18	R&D Video Technician	Designing systems for remote and low-latency live streaming services
P19	Set Designer	Involved during the preparation of indie film & tv sets
P20	Video Consultant	Involved by companies to design video strategies and oversee their production

#### Procedure

The semi-structured interviews were conducted using videoconferencing software, in either English or Italian. Each of the interviews took around 60 minutes. The interviews were recorded and later transcribed. The responses from the non-English speaking participants were translated into English. The interviews were split into two different parts.

##### Part 1 - user requirements gatherings

The first part gathered a broad view of the participants’ perspectives on their challenges during the pre-production phase of an AV production, and specifically when they are required to shoot in a real-world location. In the first part of the interview, four standard ethnographic-oriented questions were used with participants in order to learn more about the participants and their background. Participants were asked about their job title, years of experience, previous projects, and challenges faced in the making of their projects:

1. *“Could you briefly tell me more about your background and your role today?”*
2. *“What’s your experience and knowledge about immersive technology?”*
3. *“Which are your challenges when doing pre-production for a project?”*
4. *“Which are your challenges when dealing with location scouting?”*

The fifth question:

5) *“How can immersive technology such as VR and AR solve some of these challenges?”*

was the core question and served as a blank sheet where participants could freely envision ways of taking advantage of immersive technologies.

##### Part 2 - evaluation of the initial solution proposed

The second part of the interview was dedicated to presenting an initial system design diagram, previously and independently designed by the researcher. After the presentation of the proposed solution, participants provided their feedback, proposed new ideas, and asked questions to gain a deeper understanding of the design solution showed.

#### Initial design solution proposed

Having identified location scouting as a key phase in the AV pre-production process, a new workflow has been ideated, taking advantage of state-of-the-art, affordable, easy-to-use, and accessible consumer immersive technologies.

**Fig. 5:**
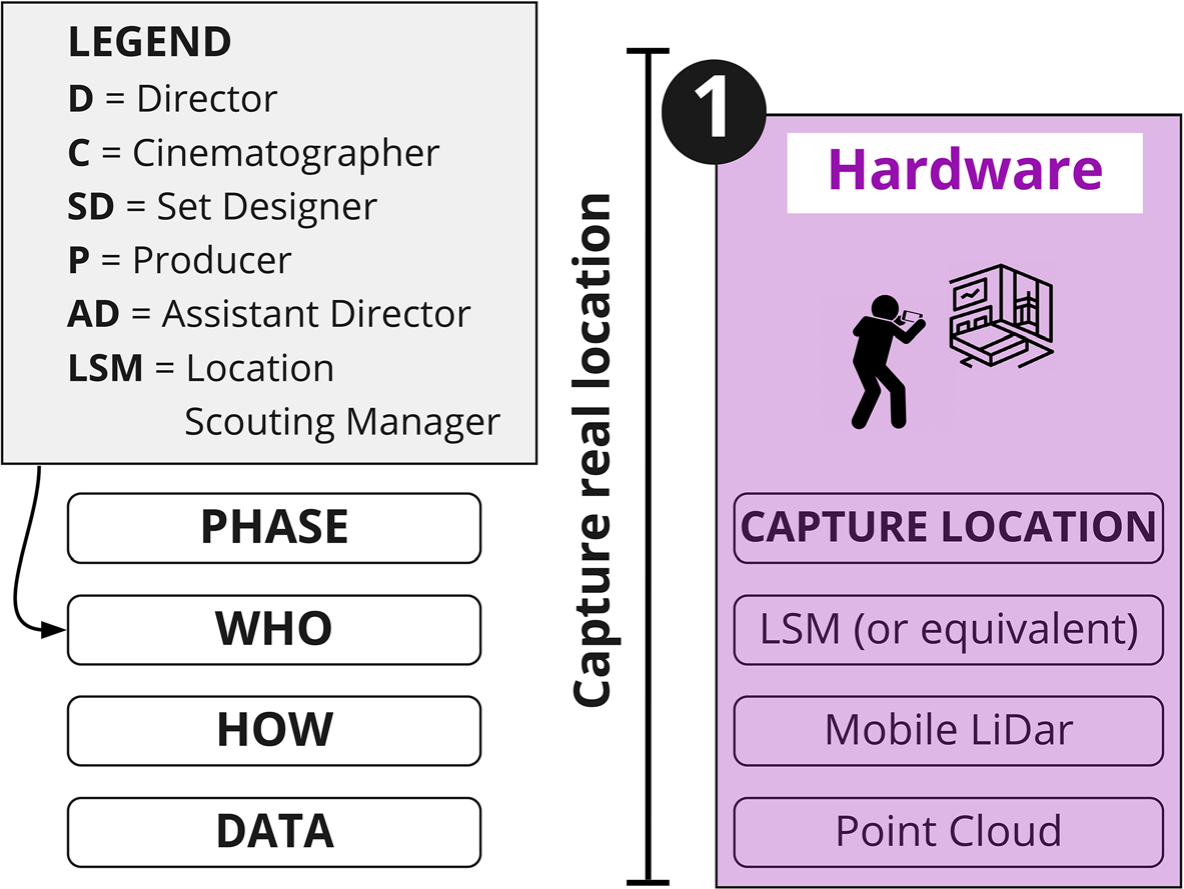
Capture phase. Legend: D=Director; C=Cinematographer; SD=Set Designer; P=Producer; AD = Assistant Director; LSM = Location Scouting Manager

As shown in Fig. 5, this workflow starts with the ‘capture’ of the set location, choosing between two 3D capturing techniques: photogrammetry and LiDAR (‘Light Detection And Ranging’). Both techniques can currently be employed using existing consumer mobile devices such as smartphones (iPhone 12) and tablets (iPadPro2020). Scanning and photogrammetry applications such as ‘SiteScape’ [33] ‘Polycam’ [26], and ‘Disaplay.land’ [39], available on the App-Store (iOs) and Play Store (Android), make this step affordable, quick, and user-friendly. The data generated after this capturing phase needs to be processed in order to generate a 3D mesh of the captured location. This step is usually automated via a relevant app.

**Fig. 6:**
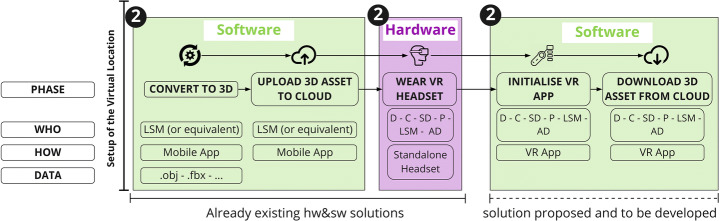
Setup Phase

**Fig. 7:**
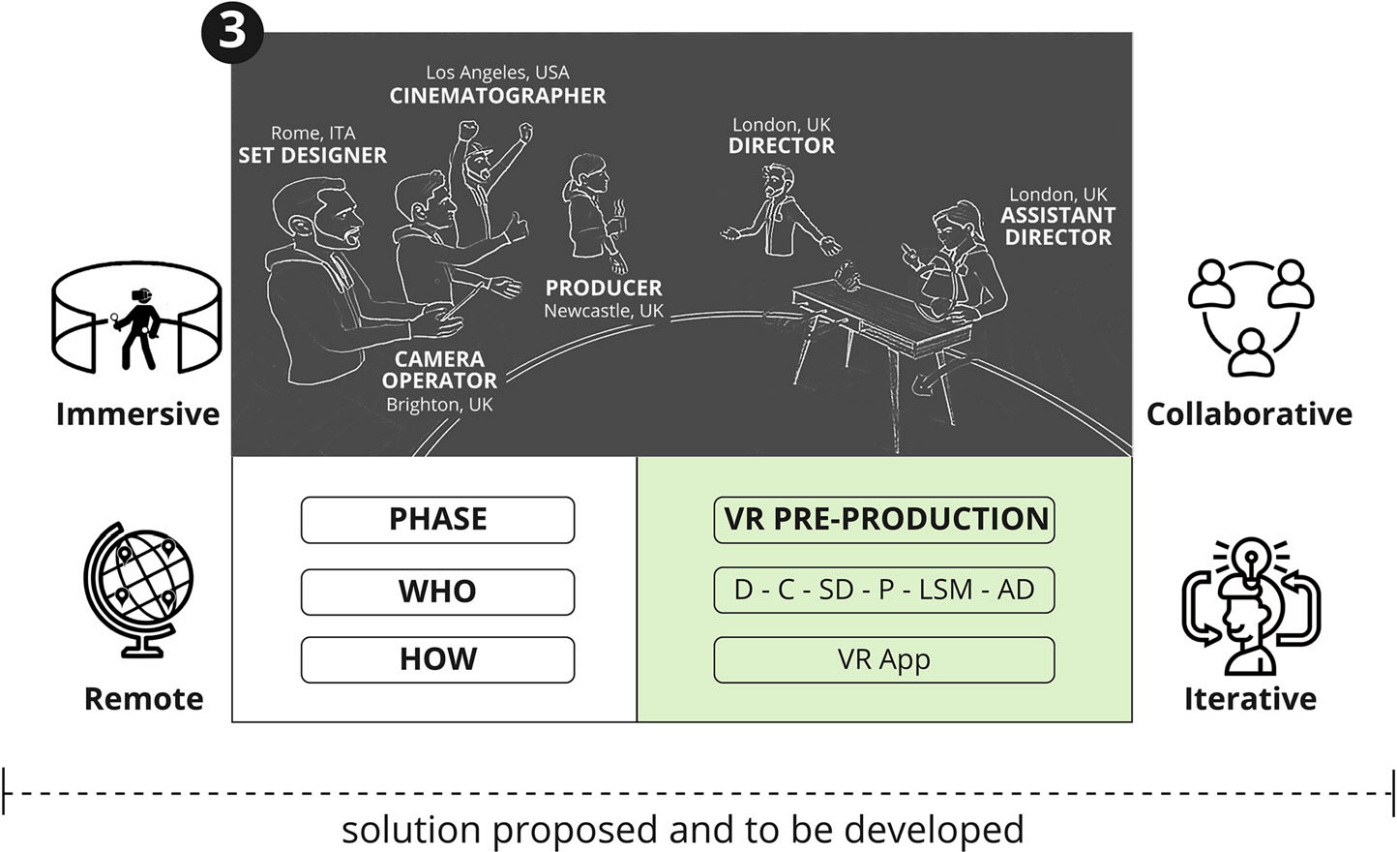
VR Pre-Production

**Fig. 8:**
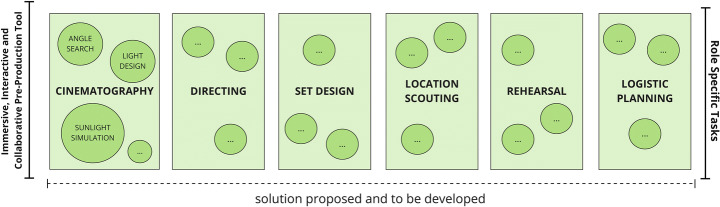
Role specific tasks

**Fig. 9:**
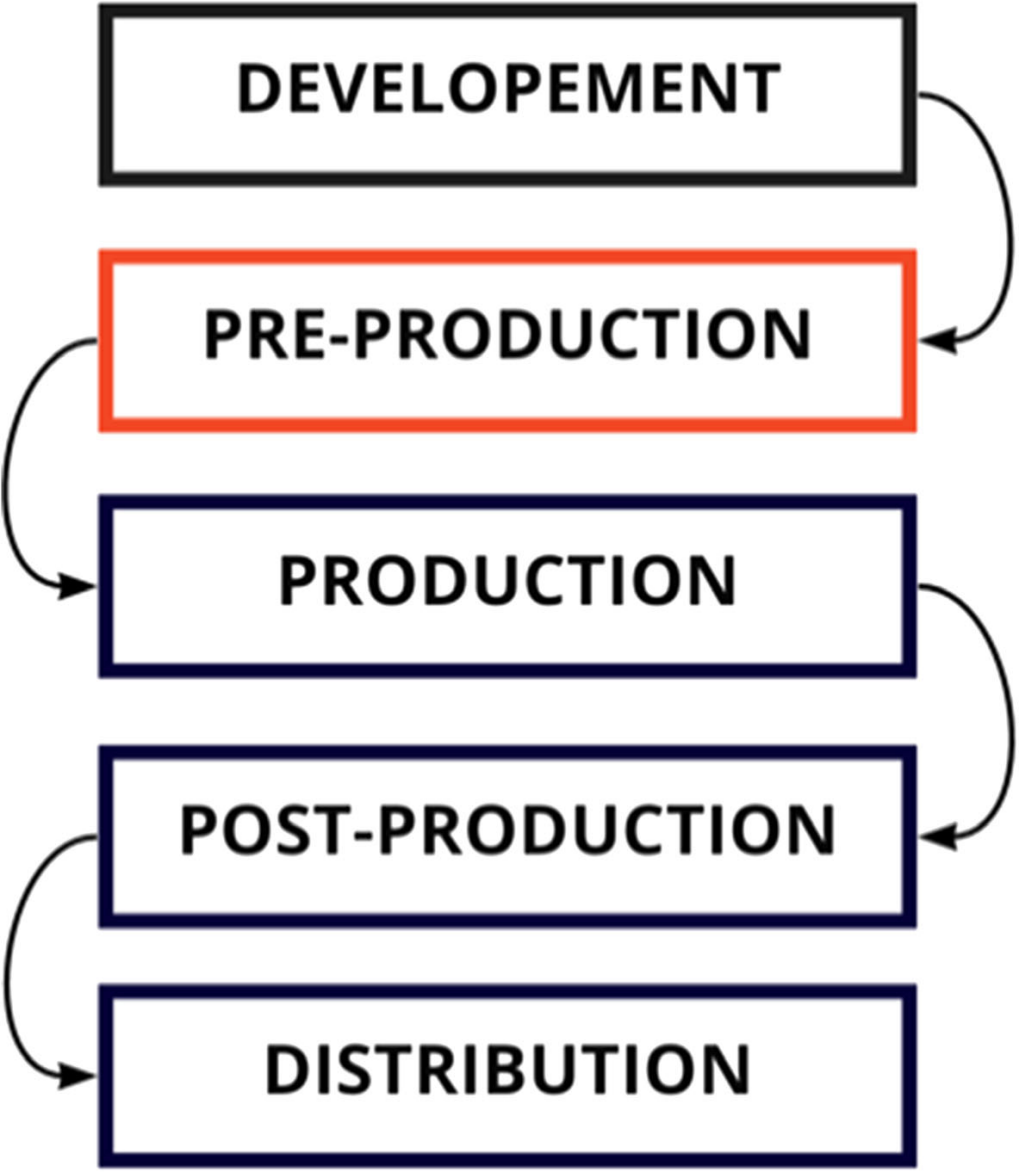
Traditional linear AV workflow, as opposed to the proposed pre-production design solution

As part of the framework that is being proposed, once the 3D location asset has been generated, it can be imported into a future VR application allowing users to immerse themselves in the digital replica of that location, inspired by the Digital Twin concept [23]. The user will then be able to navigate and interact with the virtual location and start pre-producing the AV project Figs. 6, 7, 8 and 9.

This will involve work on different aspects, such as choice of camera position and lenses, selection of camera movements, lighting setup, actor blocking (a phase where the Director decides actors’ positions and movements), rehearsal, set design, and logistics. Dedicated tools will be designed for each task.

### Findings

After completing the interviewing process, data was analysed following the six steps of analysis presented by Braun [8]. The main goals of this phase were to identify the most relevant and common themes emerging from the data gathered, shown in Table A, and to validate and integrate the virtual location scouting design solution presented in the second part of the interview. The thematic analysis was conducted using Nvivo12 [27].

## Discussion

The data collected from the participants allowed the identification of those daily challenges that are common to all the participants involved in independent AV productions and those that are role-specific.

### Common challenges

The making of a professional AV production requires coordination of contributions from people with different skillsets, from pre-production to post-production.

For this reason, efficient *communication* across departments throughout the process was identified as key by most participants. The way needs, issues, tasks, and ideas are communicated is crucial no matter whether the communication is about sharing technical information on where to park the truck outside the location or about creative ideas such as the effects that need to be achieved through lighting.

*Preparation* is crucial in order to avoid unexpected problems during shooting, generally the most expensive phase of the entire process, and to enable exploration of different creative solutions.

*Pre-vi﻿suali﻿sation* i﻿s a combination of *communication* and *preparation*. Participants pointed out how *pre-visualisation* is extremely useful when they are working in the pre-production phase. Even a rough visual representation of how the creative roles, generally the Director, envision the scene, can greatly facilitate communication across the whole team. This visual information is useful not only to the Director’s creative collaborators such as the Cinematographer and Costume Designer, but also to more organisational roles such as the Producer or Assistant Director who are required to plan and manage the production.

### Role-specific challenges

- Directors

When approaching a project, Directors are responsible for identifying the best way of visually translating what is written on the script. This involves coordination of different roles to help Directors achieve their vision. However, as P10 (Cinematographer) pointed out: “not everybody speaks the same language”. The same words on a script can be interpreted differently by different readers. For this reason, aligning all crew members to the same vision is one of the main challenges for Directors throughout the production process, and particularly during pre-production.  Visual materials such as mood boards are currently used to address this challenge. This can be combined with the creation of detailed storyboards for sharing proposed solutions. These approaches can also be useful when Directors are looking for funding and need to pitch their project to potential funders.

b) Cinematographers

Crucial to recreating the best visual look for the project, Cinematographers propose the most appropriate creative and technical solutions to Directors. They have a keen eye for images and deep knowledge of what technical gear is required (e.g. cameras, movements, lenses, filters, and lights) to fulfill the Director’s vision. Despite this, it is not always possible for them to anticipate which filming and lighting equipment are best employed. This is even more relevant to aspiring and new Cinematographers with limited experience. This attitude may lead them to recreate lighting setup or camera movements used in previous projects to reduce risk of mistakes and delays in the production,  thereby limiting their creative potential. Cinematographers are involved in the location scouting phase and during the subsequent ‘technical scouting’ phase during which they become aware of technical information such as ceiling height, availability of electrical power sources, and the presence of reflective surfaces, just to name a few.

iii) Producers

Producers have to organise and oversee the whole process, including budgeting the script and securing funding, arranging logistics during production, identifying a facility where to start post-production, and ensuring the final delivery. Throughout the entire workflow, a good Producer tackles in advance those problems that may arise in later phases of the making process. This includes getting permission for shooting on public streets, ensuring catering has taken into account food allergies or specific requests, and writing the daily call sheet to estimate the budget needed to make the project happen. Producers have several tasks to complete before the beginning of the shooting phase, and careful planning can make the difference between failure and success.

“Any problem that gets brought up on-set is infinitely more expensive than a problem that was brought up beforehand, because beforehand it’s just a problem that doesn’t have a value to it [...] You can’t foresee all problems, but it kills you if there’s a problem you could have foreseen.”

— P06, Producer

Study participants covering the Producer role pointed out that they need to communicate with all crew members for logistical reasons, understand their specific needs and evaluate the best solutions. As explained by P07 (Producer), their priority is to keep everyone on the same page because “streamlining communication tends to kind of always be an issue and something gets lost in translation sometimes”. Before and during the production phase, Producers have to share a variety of documents and be sure that everyone has a clear idea of what is expected of them. Like Directors and Cinematographers, Producers are deeply influenced by the nature of the chosen location. Depending on the findings from location and technical scouting, they will organise the production phase differently.

### Participants’ views ﻿on the use of immersive technologies in the AV pre-production process

When participants were asked to express their views ﻿on how immersive technologies such as VR and AR might help them address﻿ their challenges, they explained that they could help them save time and improve use of financial resources, as well as enhance their creativity. The main benefits they expect are listed below.

1. *Avoid travelling and save time during location auditioning*

The framework that is proposed in this article will make it possible to experience locations remotely within immersive environments. It is considered to be useful to support the decision-making process during a preliminary phase called ‘location auditioning’. In this phase many options are considered. The proposed solution will avoid physical travel from location to location, thereby saving time, optimising use of financial resources, and reducing the environmental footprint. Having the possibility to immerse oneself in the virtual location and explore it remotely will overcome several limitations of traditional 2D photos currently used to pre-select the possible locations.

“If I could wear the headset and be back in the room, where I took the photographs...go home and instead of having only my memory or photo in two dimensions being able to see the space in three dimensions I think it would make me think much more clearly about where and how I want to place the equipment.”

— P12, Cinematographer

The participants felt that physically scouting the chosen location at least once, following the virtual screening, is still vital, which suggests that the proposed solution could be integrated within existing workflows without necessarily replacing traditional processes in their entirety.

2) *Remote Collaboration*

Several participants speculated that virtualising the location scouting process would also favour collaboration across team members based in different geographical locations.

3) *No more constraints*

During the interview, a participant pointed out the following:

“There are a lot of independent productions that don’t allow time for location scouts, which I really don’t like and then I just get thrown into it on the day, and I’m not sure.”

— P10, Cinematographer

The new workflow that is proposed in this article will allow professionals to access a digital capture of the selected location at any point in time. This will in turn enable them to spend more time exploring the virtual space, thereby facilitating the generation of creative output.

4) *Virtual auditioning of props and costumes*

P06 (Producer) commented on the benefits of virtual exploration ahead of physical location scouting in terms of time saving and more efficient use of financial resources, e.g. in relation to better choice of dresses and furniture to be rented.

“The Director hopefully could sit there and look at and go - I don’t like it. I don’t like it. Okay, this is the one that we should run because I like the way it looks in the actual scene.”

— P06, Producer

5) *Facilitating logistics*

Having the possibility to do location scouting and technical scouting virtually, many logistical and technical questions may be addressed by the head of the different departments and usually answered by Producers. Some of the most common questions that need to be answered before shooting day in order to help the production to run smoothly are the following: *“Where to park the truck?”, “Where to store the equipment?” “Where are the power sources?” “Where to create a holding area?” "Where to create the production office?”* (P06, Producer). These are some of the most common questions that need to be answered before shooting day in order to help the production run smoothly.

6) *Lighting setup pre-visualisation*

When planning the lighting setup, the proposed solution is expected to be useful to pre-visualise how a certain light model or disposition will affect the look of the picture (e.g. how a hard light or soft light would illuminate the actors’ faces). Similarly, pre-visualising the impact of sunlight coming directly or bouncing from a window is crucial and can affect the Director’s and Cinematographer’s decisions. Being able to simulate how sunlight will influence the lighting of the scene on a specific day and time would be a useful addition because *“sometimes during location scouting, you’re not necessarily there at the exact right time of when you intend to shoot”* (P10, Cinematographer). Some Cinematographers currently rely on mobile apps such as Helios Pro [10] that are non-immersive in nature and therefore have limitations.

7) *Camera movement pre-visualisation*

Taking advantage of VR, it will be possible to pre-visualise complex camera movements achievable with expensive gear such as lights, cranes, and dollies, and then evaluate their use in production if considered appropriate for fulfilling the Cinematographer’s and/or the Director’s vision. Having such an opportunity will therefore help professionals at the time of renting/purchasing the equipment required for production because they had a chance to test it virtually in advance.

8) *Pitching and fundraising*

The proposed framework has the potential to facilitate the creation of a storyboard and/or a teaser to pitch to potential funders and investors. In this regard, P12 provided the following example:

“Yes, let’s take in example the idea of using a crane...-Guys, this is how it would look like if we take the cherry-picker and do the shot from top to bottom. To do so we have to spend 1200£ or more because we need the crane and 3 operators. That’s it. What do we do? Do we spend them or not?”

*—* P12, Cinematographer.

Also, as explained by P08, this application can have the following benefit:

“Empower beginning or mid-career Directors who want to be ambitious and are trying to push the limits and are very creative, very innovative and are looking at creating something that doesn’t exist and they might not have the resources for. I think it’s for visionaries.”

— P08, Director

9) *Communication among crew members*

P12 (Cinematographer) gave an example of his relationship with the Key Grip, who is the professional in charge of positioning and setting lights on set to illuminate the scene as desired by the Cinematographer:

“Sometimes you explain all the work to the key grip and he don’t remember everything you explained him, at the end of each scene you have to explain it all over again. And sometimes you have to talk to the Director, to the client, you are busy with other things.”

— P12, Cinematographer

This process is usually implemented verbally or using a piece of paper where a rough scheme has been drawn, but it is not rare for one of the people involved in the conversation to forget some details about the setup. The participant speculated that providing his Key Grip with a VR device on set, so they can immerse themselves in the pre-visualisation of the project with all the lighting setup established in advance, would lead to better and more effective communication and would reduce delays and risk of mistakes. A similar principle can also be applied in other contexts. One example is the collaboration between the Cinematographer and Director, where moving virtual cameras, cranes, lights, and props can be done more efficiently and transparently.

### Evaluation and iteration of the solution proposed through participatory design

**Fig. 10:**
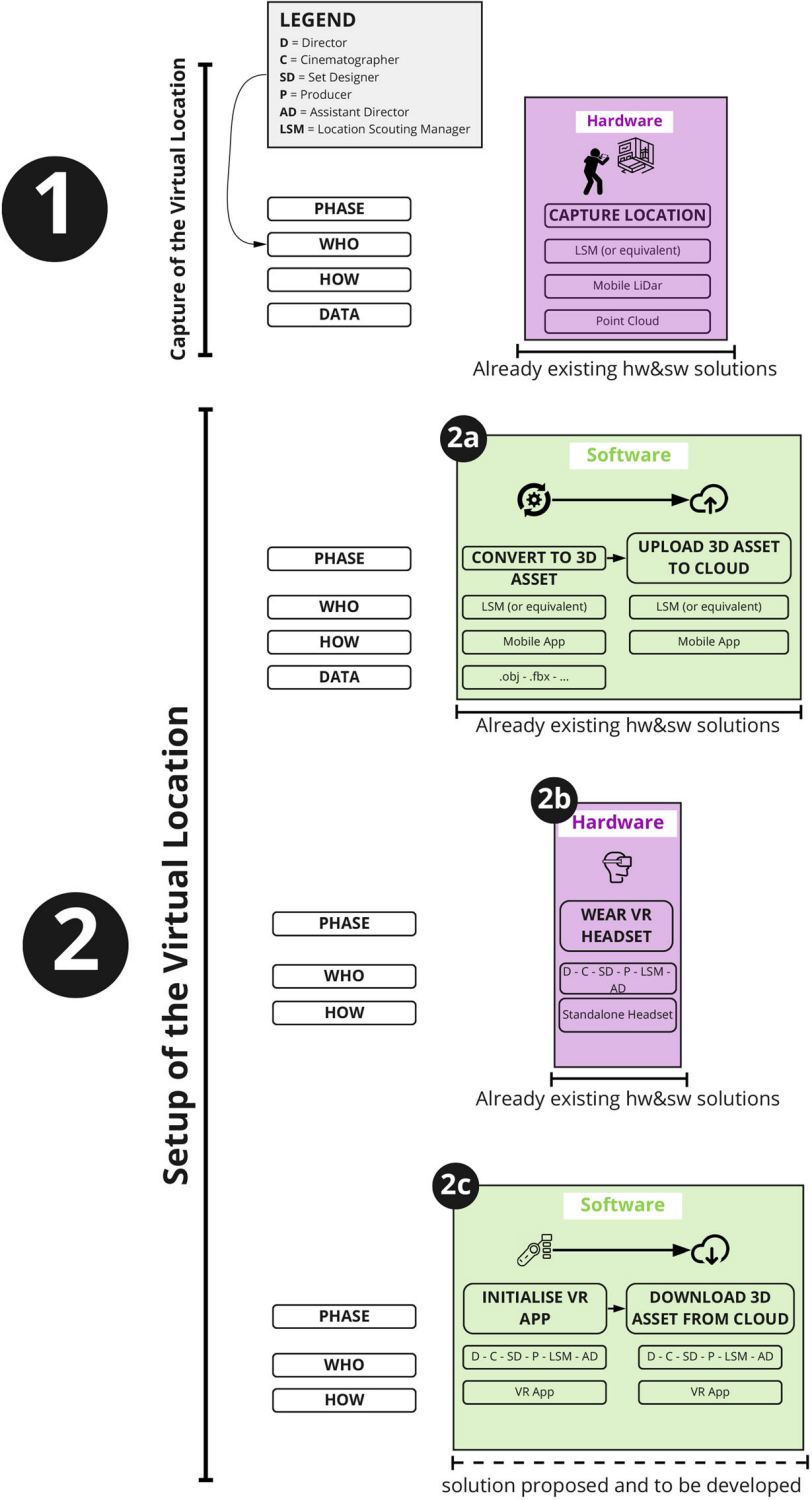
System Design Diagram incorporating ideas and feedback (Phase 1 and Phase 2)

**Fig. 11:**
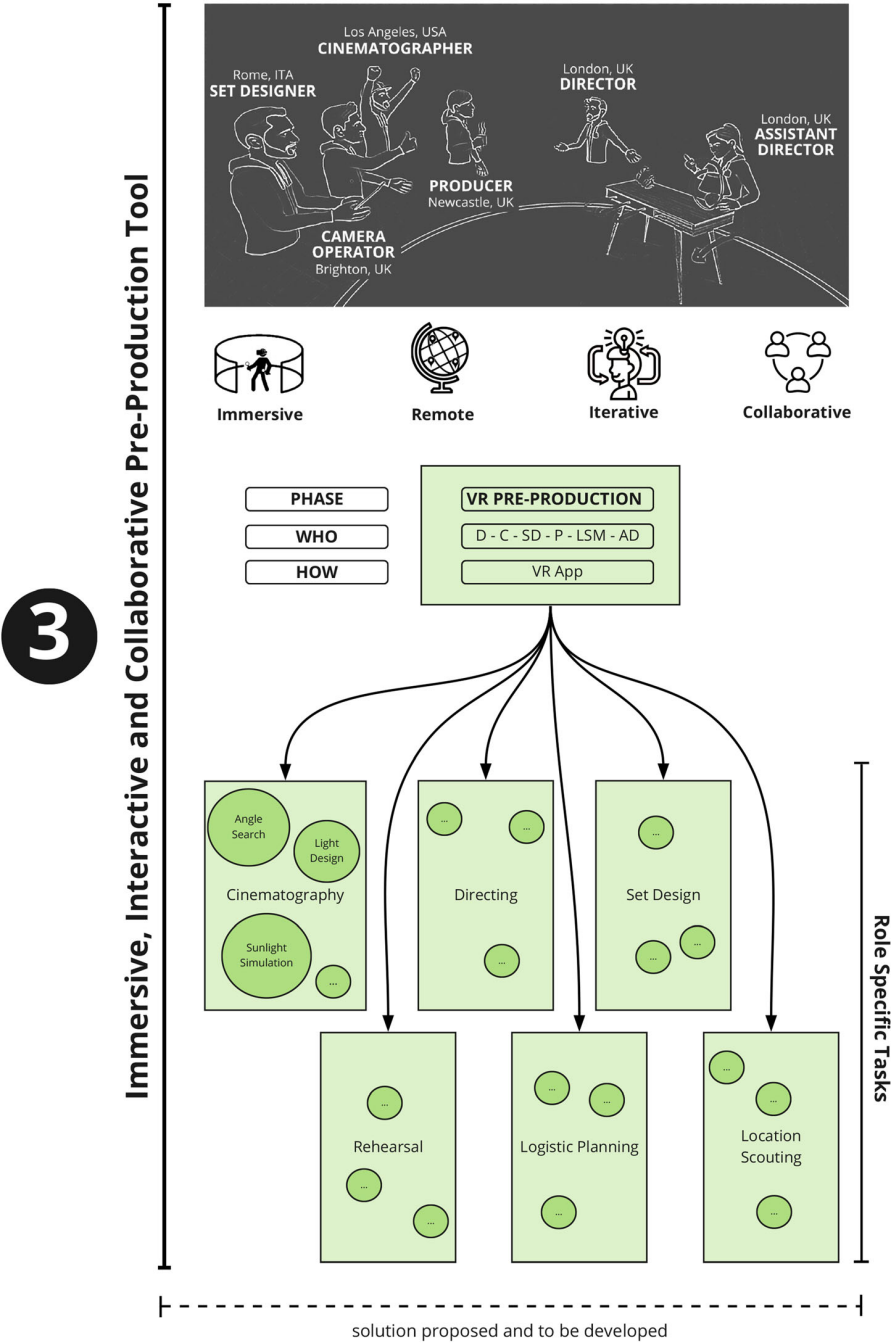
System Design Diagram incorporating ideas and feedback (Phase 3)

Participatory design principles have been used for evaluation within an iterative design cycle throughout the interviewing process. The positive feedback and comments received from all participants on the initial design solution have been used to add elements to the initial design and to strengthen the concept as a whole. The result of the integration is shown in Figs. 10 and 11 which are conceptually part of the same diagram.

**Fig. 12:**
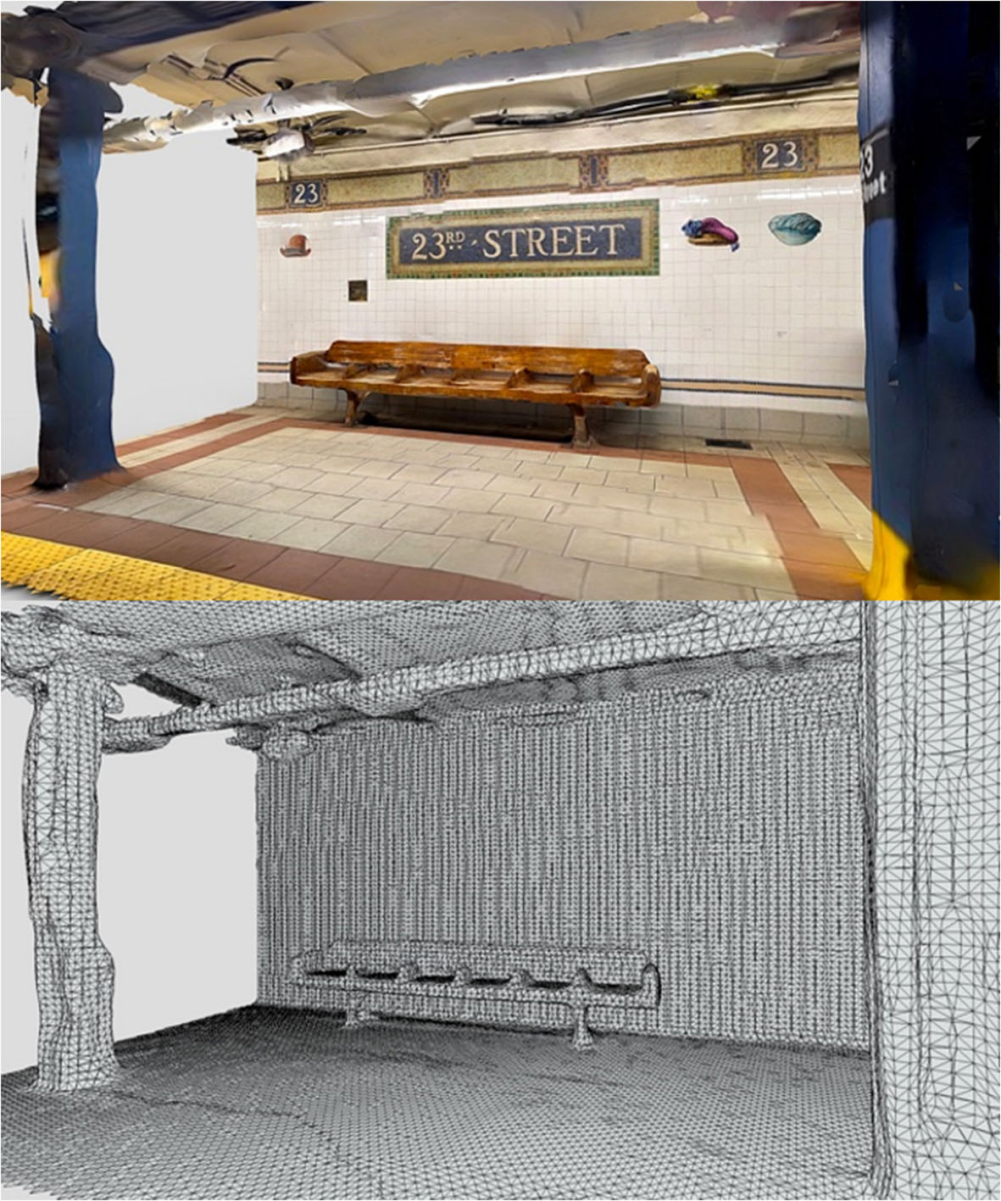
Final output after capturing data with iPhone 12 Pro’s LiDAR and processing with Scaniverse app (top). Wireframe view (bottom). Images from [1]

Visual quality of the digital location captures, obtained via either photogrammetry or LiDAR scans (Fig. 7), was highlighted as critical by participants during the interviews. Similar remarks were made about the integration of all other relevant virtual elements in the scene at later stages of the production process Fig. 12.

Due to the trade-off between affordability and output quality of the available digital capture mobile hardware and software, it was necessary to evaluate whether this solution could be beneficial for film professionals. Most participants thought that having the possibility of moving freely in a 3D space (thanks to the 6DoF allowed by current standalone VR headsets) is a greater benefit compared to having a high-fidelity representation of the location without the freedom to explore the space around them.

A remark was also made by Cinematographers about the quality of virtual lighting simulation. Simulating reflections and proper lighting has actually always been a challenge in real-time computer graphics. Participants pointed out the potential detrimental effect of inconsistency between pre-visualisation and the real production scenario, particularly in terms of misleading information. Nevertheless, having the opportunity to place lighting equipment in a virtual location was considered useful in order to gain a better understanding of how much space is required for lights and other lighting-related equipment such as stands, booms, flags, and jibs.

“Even without realism, if I place a light from above, representing roughly the lighting effect, the Director can fill the gap of the lack of representation with his imagination to visualise how it would look on set in reality.”

— P12, Cinematographer

The proposed framework is intended to help different AV professionals pre-visualise locations and prepare for production. For this reason, access to high-fidelity representations is not a priority, as the pre-visualisation output is not the final output from the project.

The possibility to collaborate remotely, seamlessly and in real time, within a digital representation of the location was also considered useful by participants. To explain this feature better, they referred to ‘Google Doc’ [15], a web application which allows different people to work and edit in real-time the same textual document. This also points to the importance of security and privacy features for a future platform implementing the proposed framework, as well as to the need to set up a permissions system for edits and revision history management.

#### The importance of ease of use

An important aspect that has been emphasised by the majority of participants is ease of use of such a workflow. In their opinion, poor usability can be a deal-breaker for the proposed solution. Learning a new workflow and related software is a tedious and time-consuming process which potential users are not necessarily willing to engage with.

“I foresee there might be a gap of between an independent filmmaker wanting to use this technology and himself actually be able to manage it [. . . ] Many independent filmmakers are in a way. . . they simplify greatly, they greatly simplify things and the workflow because of the condition of the productions or budget or maybe even people. And they are okay to do so, in order to get the movie done.”

— P03, Cinematographer

“How long it would take? How complicated it is? What the learning process would be for someone who isn’t necessarily experienced in visual effects or 3D modelling?”

— P08, Director

For this reason, participants suggested that they should be actively involved in designing intuitive user interfaces when the workflow is implemented, in order to reduce the slope of the learning curve and facilitate rapid progression for all users.

#### The importance of already existing solutions

An aspect that surprised participants was the present availability of key components of the proposed solution, thanks to existing price-wise competitive hardware and software, affordable to independent productions with limited resources. Making them aware of such solutions contributed to increasing their interest, and they have expressed an interest in being involved as beta testers of the first version of the prototype.

#### The importance of uniqueness

Another aspect highlighted by P03 (Cinematographer) is that what is often most thrilling and interesting is the unexpected. When working on observational documentaries, P03 explains how such productions are rich of unique and fortuitous moments such as the genuine reaction of the people interviewed and spontaneous events happening in the area while shooting. At the same time, he stated:

“Even if it’s a documentary, there could be sequences that are more constructed and even slightly rehearsed. And this tool can help that too. So it can be used on documentaries as well. I wouldn’t rule it out.”

— P03, Cinematographer

## Conclusions

Through qualitative analysis of interview data from AV professionals, the key daily challenges faced by Directors, Cinematographers, and Producers during pre-production have been identified, and the anticipated benefit of adopting immersive technologies﻿ in AV production has﻿ been determined. Based on the participant feedback and comments, this article has proposed a workflow for embedding Virtual and Augmented Reality technology throughout the AV production process, with an emphasis on location scouting and pre-production. The user requirements for the development of a first generation of prototypes have been collected and discussed.

## Future work

Further extension to this research will include testing existing commercial hardware and software tools. The objective will be to identify the most suitable devices for integration in the first part of the workflow, and to capture the digital replica of a location. An additional user experience study will be carried out, together with user interface development, building on co-design workshops with relevant stakeholders. Once a working prototype is available, it will be investigated in relation to its use in a real-world independent production, thereby providing the stakeholders with all the information and equipment they need to implement the workflow autonomously. This﻿ will make it possible to evaluate how the prototype impacts the quality of work and to quantify the anticipated time and cost savings as a result of its adoption. The proposed workflow for capturing and experiencing virtual locations can also be appealing to private location scouting agencies, as well as to public film institutions,  that will be able to create their own archives of location digital replicas to present to AV productions.
